# Psoriasis: Immunological and genetic blueprints driving pathogenesis and potential for personalized therapies

**DOI:** 10.22038/ijbms.2025.85335.18442

**Published:** 2025

**Authors:** Mohamed J. Saadh, Omer Qutaiba B. Allela, Radhwan Abdul Kareem, Gaurav Sanghvi, G. PadmaPriya, Rishabh Thakur, Mukesh Kumari, Sofia Gupta, Kakhramon Khaitov, Hayder Naji Sameer, Ahmed Yaseen, Zainab H. Athab, Mohaned Adil

**Affiliations:** 1 Faculty of Pharmacy, Middle East University, Amman, 11831, Jordan; 2 College of Pharmacy, Alnoor University, Nineveh, Iraq; 3 Ahl Al Bayt University, Kerbala, Iraq; 4 Marwadi University Research Center, Department of Microbiology, Faculty of Science, Marwadi University, Rajkot-360003, Gujarat, India; 5 Department of Chemistry and Biochemistry, School of Sciences, JAIN (Deemed to be University), Bangalore, Karnataka, India; 6 Centre for Research Impact & Outcome, Chitkara University Institute of Engineering and Technology, Chitkara University, Rajpura, 140401, Punjab, India; 7 Department of Applied Sciences-Chemistry, NIMS Institute of Engineering & Technology, NIMS University Rajasthan, Jaipur, India; 8 Department of Chemistry, Chandigarh Engineering College, Chandigarh Group of Colleges-Jhanjeri, Mohali140307, Punjab, India; 9 Department of Dermatovenerology, Pediatric Dermatovenerology and AIDS, Tashkent Pediatric Medical Institute, Bogishamol Street 223, Tashkent, 100140, Uzbekistan; 10 College of Pharmacy, National University of Science and Technology, Dhi Qar, 64001, Iraq; 11 Gilgamesh Ahliya University, Baghdad, Iraq; 12 Department of Pharmacy, Al-Zahrawi University College, Karbala, Iraq; 13 Pharmacy College, Al-Farahidi University, Iraq

**Keywords:** Genetic predisposition, Immunopathogenesis, Inflammation, Personalized medicine, Psoriasis

## Abstract

Psoriasis is a long-lasting inflammatory skin condition that impacts millions globally. The occurrence of this disorder differs significantly across various areas, resulting from a complex interplay of genetic and environmental influences. In psoriasis, the pathogenesis represents a complex interaction of innate and adaptive immunity that plays a significant role in the disease manifestation process. Many genetic factors predispose to psoriasis, which is considered a polygenic disease. Several genes concerning pathways like NF-κB and PI3K/Akt that modulate the amplification of inflammatory response and keratinocyte dysregulation have been elaborated in the light of their differential expression, susceptibility loci, and polymorphisms. Such genetic insights could open a whole new avenue for precision medicine in which biomarkers and gene-targeting therapies are promising options for personalized treatment. This review emphasizes the need for complex investigations into psoriasis, from molecular mechanisms to clinical manifestations, to bridge the gap between basic research and therapeutic development by furthering the understanding of psoriasis and paving the way for innovative treatments addressing skin lesions and systemic effects.

## Introduction

Psoriasis is a chronic inflammatory skin condition characterized by an abnormal immune response that leads to the hyperproliferation of keratinocytes, disrupting the skin barrier. This condition manifests globally, affecting a diverse population across various demographics. The prevalence of psoriasis varies by geographical region and ranges from 0.11% to 1.58% (1). It is estimated that around 125 million individuals globally are affected by psoriasis (2). The condition manifests in different forms, including plaque, guttate, inverse, pustular, and erythrodermic psoriasis; however, psoriasis vulgaris is the most common type, accounting for 85–90% of all psoriasis cases (3). Psoriasis vulgaris is characterized by developing well-defined, raised, red, and scaly oval-shaped plaques resulting from abnormal cell growth, differentiation, and immune cell response (4). In addition, psoriasis has been linked to a range of comorbidities, including psoriatic arthritis, hypertension, diabetes mellitus, and cardiovascular diseases, among others (5). The implications of psoriasis for society and healthcare systems are significant, given the associated healthcare costs and impact on quality of life. The etiology and pathogenesis of psoriasis present a complex and multifaceted phenomenon yet to be fully comprehended. Psoriasis is currently recognized as a disease driven by the immune system, resulting from an intricate interplay between innate and adaptive immune responses. This interaction results from the complex relationship between genetic predisposition and environmental risk factors (6). Recent advancements in understanding the molecular mechanisms associated with psoriasis have been noteworthy (7, 8). Genome-wide studies have identified novel genes linked to this condition and have demonstrated that multiple genes contribute to the disease’s pathogenesis. These findings hold significant implications for therapeutic approaches (9). This review intends to outline the existing evidence regarding the immune mechanisms and genetic influences that play a role in the development of psoriasis.

### Immunopathogenesis, cellular interactions, and cytokine dynamics in psoriasis

Psoriasis is a complex skin disorder characterized by excessive skin cell production and immune cell infiltration into the dermis. This process involves intricate interactions among keratinocytes, immune cells, and other cell types within the skin. Moreover, various other cells are found in the skin. In the last twenty years, psoriasis has gained recognition as a disease driven by immune cells, with keratinocytes acting as the agents that carry out immune cell functions. The IL-23/IL-17 pathogenic axis has emerged as a key contributor to psoriasis, driving the pathophysiological changes within the skin (10).

Dendritic cells are widely recognized for their essential role in the initial phases of psoriasis (11). These specialized cells play a crucial role in presenting antigens; nevertheless, the processes that lead to their activation in the context of psoriasis are still not fully understood. One suggested mechanism is identifying antimicrobial peptides (AMPs) that keratinocytes release following an injury and are typically found in higher amounts in psoriatic skin. AMPs extensively studied in relation to psoriasis include LL37, β-defensins, and S100 proteins (12). The peptide LL37, called cathelicidin, has been associated with an adverse role in the progression of psoriasis. Specifically, it has been observed that when keratinocytes are damaged, they release LL37, which subsequently binds to self-genetic material from other damaged cells, forming complexes. These LL37-bound complexes have been shown to stimulate Toll-like receptor 9 (TLR9) in plasmacytoid dendritic cells (pDCs) (13). The activation of pDCs is critical for the initiation of psoriatic plaque formation. This process is marked by the release of type I interferons (IFN-α and IFN-β). The type I interferon signaling pathway facilitates the phenotypic development of myeloid dendritic cells (mDCs). It has been linked to the differentiation and function of Th1 and Th17 cells, which includes the generation of IFN-γ and interleukin (IL)-17, respectively (14).

The interaction between LL37 and DNA complexes stimulates pDCs through the TLR9 pathway. However, the interaction between LL37 and RNA complexes has been found to stimulate pDCs via the TLR7 pathway. Moreover, LL37–RNA complexes influence mDCs via the TLR8 signaling pathway (15). Once activated, mDCs move to the lymph nodes that drain the site and produce cytokines such as tumor necrosis factor (TNF)-α, interleukin (IL)-23, and IL-12. The synthesis of IL-12 and IL-23 is vital for controlling the differentiation and proliferation of T-helper (Th) 1 and Th17 cell subsets, respectively. Additionally, slan+ monocytes, key pro-inflammatory cells in psoriasis skin lesions, react to LL37–RNA activation by producing significant amounts of TNF-α, IL-12, and IL-23 (16).

**Figure 1:**
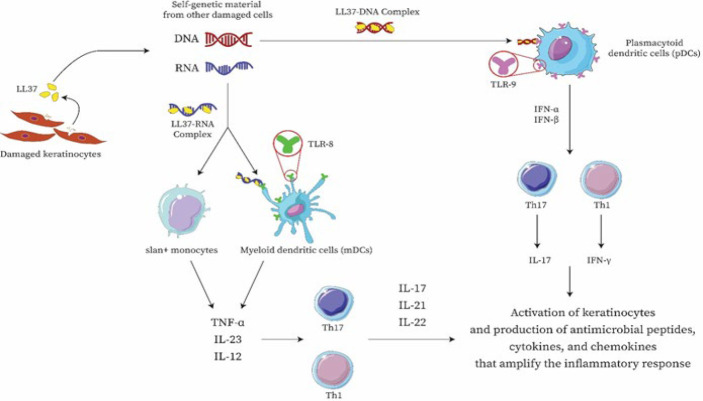
Role of LL37 in the progression of psoriasis

Upon activation, pDCs enhance the maturation process of mDCs and stimulate the secretion of TNF-α, IL-12, and IL-23. This process activates Th1 and Th17 cells, releasing inflammatory cytokines like TNF-α, IL-17, IL-21, and IL-22. Consequently, keratinocytes become activated, especially in response to IL-17, producing antimicrobial peptides, cytokines, and chemokines that amplify the inflammatory response (17). Figure 1 briefly illustrates these complex interactions. 

Keratinocytes are crucial in both the beginning and upkeep stages of psoriasis. As part of the innate immune system, keratinocytes respond to a range of stimuli. When under stress, keratinocytes emit self-nucleotides and antimicrobial peptides, promoting the activation of pDCs. Consequently, mDCs are activated and matured, leading to the production of IFN-α, IFN-γ, TNF-α, and IL-1β (18). Besides their function in the initiation phase, keratinocytes also act as amplifiers of psoriatic inflammation throughout the maintenance phase (19). In response to pro-inflammatory cytokines, keratinocytes may proliferate significantly and produce a variety of chemokines, including CXCL1/2/3, CXCL8, CXCL9/10/11, CCL2, and CCL20. These chemokines play a crucial role in drawing different types of leukocytes, including neutrophils, Th17 cells, dendritic cells, and macrophages. In addition, keratinocytes can generate antimicrobial peptides such as S100A7/8/9/12, hBD2, and LL37, which contribute to the activation of innate immunity and other inflammatory mediators that enhance inflammation. In addition, keratinocytes are crucial for tissue reorganization as they activate and proliferate endothelial cells, deposit extracellular matrix, and work with fibroblasts and endothelial cells (20). The interplay between keratinocytes and immune cells, especially Th17 cells, leads to psoriasis, which is marked by excessive proliferation and abnormal differentiation of keratinocytes, enlarged and overdeveloped blood vessels, and the infiltration of inflammatory cells (21).

Growing evidence indicates a notable rise in the RNA expression levels of IL-23 within psoriatic lesions (22). The concentration of IL-23 protein in psoriatic lesions is markedly higher than in the unaffected skin, thus implying that the pathogenesis of psoriasis is linked to IL-23. A cellular receptor complex facilitates the interaction between the IL-23 protein and CD4+ helper T cells, specifically Th17 cells. Consequently, Th17 cells produce IL-17, an essential cytokine that plays a key role in developing psoriasis by activating various intracellular signaling pathways. This process highlights the essential role of IL-23 in the progression of psoriasis (23). Given the role of the IL-23/IL-17 axis in psoriasis pathogenesis, biological therapies targeting these cytokines have been developed to improve patient outcomes. Ustekinumab, an IL-23 inhibitor, has been demonstrated to significantly improve psoriasis symptoms by targeting the IL-23/Th17 axis, which plays a key role in the disease’s immunopathogenesis (24). Furthermore, the Inhibition of IL-17 with drugs, including brodalumab, ixekizumab (IXE), and secukinumab (SEC), has been shown to provide a robust clinical response and a significant improvement in psoriasis patients’ quality of life (25).

The elevated expression of tumor necrosis factor-alpha (TNF-α) in psoriatic lesions is a key driver of the inflammatory process underlying psoriasis, as demonstrated by the effectiveness of TNF-α-targeted therapies (26). TNF-α is produced by various cell types involved in psoriasis progression, including keratinocytes, dendritic cells (DCs), neutrophils, mast cells, natural killer T (NKT) cells, and T-helper cells (Th1, Th17, Th22) (27). Together with IL-17A, TNF-α plays a central role in regulating the expression of cytokines and keratinocyte genes, thereby modulating keratinocyte function in psoriasis (28). 

CCL20, known as Macrophage Inflammatory Protein-3α (MIP-3α) or Liver and Activation-Regulated Chemokine (LARC), is a chemokine that is particularly important in psoriasis. CCL20 is unique among chemokines because it can bind to only one receptor, C-C chemokine receptor type 6 (CCR6). The unique engagement between CCL20 and CCR6 is considered essential in developing psoriasis (29). The primary origin of CCL20 is keratinocytes, whereas CCR6 is predominantly found in dendritic cells, T cells, and Th17 cells (30). It has been discovered that keratinocyte transglutaminase 2 (TG2) can increase the expression of CCL20, attracting CCR6+ γδ T cells. Furthermore, cytokines such as IL-17A and TNF-α can increase the expression of CCL20 in keratinocytes (31).

The pathogenesis of psoriasis, particularly generalized pustular psoriasis (GPP), is predominantly influenced by the IL-36-IL-1 inflammatory axis. This axis plays a critical role in the skin’s inflammatory response, characteristic of this condition. Psoriasis is associated with the up-regulation of IL-36α, IL-36β, IL-36γ, and IL-36Ra. This enhanced expression contributes to the production of antimicrobial peptides in keratinocytes, thereby underscoring their significance in psoriasis pathology (32). The pathogenic importance of the IL-36 axis in psoriasis has been confirmed by discovering mutations in genes associated with the IL-36 signature, particularly in the IL-36RN gene (33). There is proof that sIL-36R, a soluble variant of IL-36R, can lessen psoriasis inflammation by attaching to IL-36γ and competing with IL-36R in a manner that depends on the dose (34).

IFNs play a critical role in psoriasis pathogenesis, with both type I and II IFNs acting as key mediators. Chronic viral infections that trigger psoriasis are related to type I IFNs, key cytokines. Treatments involving type I interferons frequently trigger or worsen symptoms of psoriasis or psoriatic arthritis (35). pDCs penetrate the skin and generate IFN-α in the early stages of psoriasis, establishing them as the innate producers of IFN-α (30). In psoriasis, IFN-γ influences keratinocytes, activating and synthesizing antimicrobial peptides like LL-37, cathelicidin, and β-defensins (36).

IL-6 has also been associated with psoriasis over the past two decades, but its effect remains controversial as it may have both positive and negative impacts. Previously, the expression of the IL-6 gene was considered a marker of the pathological state of psoriasis and psoriatic arthritis (37). Some studies have shown that IL-6 blockers such as tocilizumab might be particularly effective in patients with pustular psoriasis, but using these drugs requires further clinical trials (38). Research indicates that IL-6 can trigger the formation of Th17 cells, which produce IL-17 that may subsequently influence the synthesis of IL-6 by keratinocytes (28).

### Molecular mechanisms and essential signaling pathways involved in psoriasis pathogenesis

The development of psoriasis is widely understood to be caused by the abnormal death of keratinocytes in the skin. The keratinization process in psoriasis is characterized by late differentiation markers, namely pyroptosis, apoptosis, necrosis, necroptosis, and ferroptosis. Studies have indicated that genes related to pyroptosis are significantly linked to atypical keratinocyte keratinization - the process of forming keratin in skin cells (39). Pyroptosis is a type of programmed cell death that is dependent on inflammatory processes. It includes the traditional caspase-1 pathway in addition to the alternative caspase-4/5/11 pathway (40). As studies progress, it has been discovered that the non-classical route of pyroptosis mediated by caspase-11 plays a role in the progression of psoriasis (41). 

IL-1β signaling is well-known to be involved in the development of psoriasis. The differentiation and activation of IL17-producing T-cells highly depend on IL-1β. Additionally, the intensity of psoriasis and the success of treatment are linked to the protein levels of IL-1β in the affected skin (42).

A key characteristic of psoriasis is the increased presence of active, phosphorylated nuclear factor kappa B (NF-κB), which has been demonstrated to have a substantial role in developing the disease. The activation of NF-κB is recognized to promote the expression of pro-inflammatory cytokines, chemokines, and adhesion molecules, all of which play a role in the ongoing inflammation and tissue injury associated with psoriasis (43). c-Rel, a gene encoding a member of the NF-κB family, is associated with susceptibility to psoriasis. The inhibition of keratinocyte growth can be achieved through the down-regulation of c-Rel, which affects the progression of these cells through the cell cycle. IL-23 is a direct target of c-Rel, and decreased levels of c-Rel may decrease IL-23 production (44). 

The MAPK family includes p38 MAPK, ERK (extracellular signal-regulated kinase), and JNK (c-Jun NH2-terminal kinase). Studies indicate that psoriatic lesions show heightened activation of ERK1/2, p38, and JNK MAPK, which implies that the MAPK pathway plays a role in psoriasis pathogenesis (45). Angiopoietin-like 4 (ANGPTL4) is a protein that has multiple functions and is secreted by various tissues, including adipose tissue, the liver, kidney, muscle, ovary, breast, skin, and the urinary system. The metabolic and nutritional status of the organism regulates the expression of ANGPTL4. In psoriasis patients, ANGPTL4 levels are significantly higher. This protein encourages the growth of keratinocytes and triggers an inflammatory reaction via the ERK1/2 and STAT3 signaling pathways, resulting in pathological alterations linked to psoriasis (46).

**Figure 2:**
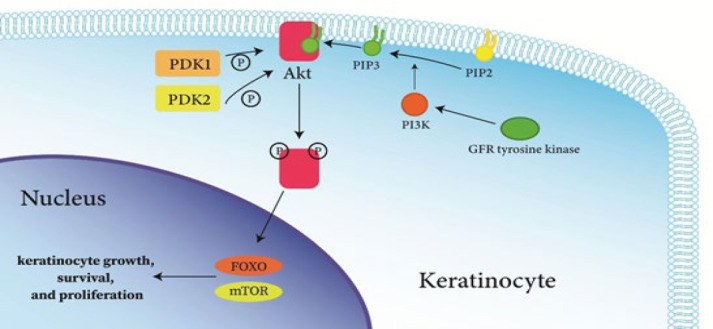
Phosphorylation of Akt by PDK1 and PDK2, its activation, and the downstream modulation of keratinocyte growth, survival, and proliferation through FOXO and mTOR

The PI3K/Akt pathway is also considered a crucial element in the development and progression of psoriasis. An increasing body of evidence suggests that the activation of this pathway, induced by either external or internal stimuli, may give rise to a range of physiological and pathological processes associated with psoriasis. As shown in Figure 2, once triggered by GFR tyrosine kinase, the PI3K enzyme converts PIP2 into PIP3 on the plasma membrane, which then binds to the pleckstrin homology (PH) domain of Akt. This binding facilitates Akt phosphorylation at threonine 308 by phosphoinositide-dependent kinase 1 (PDK1), while phosphoinositide-dependent kinase 2 (PDK2) phosphorylates Akt at serine 473. Upon full activation, Akt serves as the central molecule, translocating to the cytoplasm and nucleus to modulate the pathogenesis and progression of psoriasis through various downstream signaling pathways (47). Both forkhead box O (FOXO) and mammalian target of rapamycin (mTOR) are downstream factors of Akt and play a crucial role in regulating keratinocyte growth, survival, and proliferation. These signaling pathways are essential for maintaining the proper functioning of keratinocytes (48). 

As advancements in psoriasis research continue, it is becoming increasingly important to elucidate the genetic factors contributing to the disease. In addition to the disrupted signaling pathways that perpetuate inflammation and keratinocyte hyperproliferation, particular genes exhibit unique expression profiles closely associated with the disease’s onset, progression, and related comorbidities. Understanding these genetic determinants will enhance our ability to develop targeted therapeutic strategies and improve patient outcomes.

### Genetic fingerprint of psoriasis

The genetic landscape of psoriasis has been extensively investigated, highlighting the intricate interplay of genetic factors in the disease’s etiology and progression. While environmental triggers and immune system dysregulation are well-documented contributors, genetic predisposition is critical to individual susceptibility and disease severity. Recent genomic studies have identified significant alterations in the expression of genes associated with inflammation and immune regulation, which are pivotal in the pathophysiology of psoriasis. By delving into these genetic mechanisms, we can gain deeper insights into the cellular processes underlying the hallmark cutaneous manifestations of psoriasis, potentially informing the development of more refined therapeutic interventions.

*Genetic and pathogenic overlaps with comorbid conditions*

The intricate genetic and immunological mechanisms underlying psoriasis define its characteristic cutaneous manifestations and contribute to systemic effects that extend beyond the skin. This systemic involvement has led to the growing recognition of psoriasis as more than a skin condition, highlighting its association with various comorbidities, including metabolic disorders, cardiovascular diseases, and atherosclerosis. These connections stem from shared inflammatory pathways, genetic predispositions, and immune dysregulation, hallmarks of psoriasis and its comorbid conditions.For instance, research indicates a significant correlation between psoriasis and atherosclerosis. Specifically, individuals with psoriasis who have had the condition for more than eight years are at a heightened risk of developing atherosclerosis compared to those without psoriasis (49). Psoriasis and atherosclerosis share similar pathogenic processes. In psoriasis, myeloid dendritic cells produce IL-12 and IL-23, which drive T cells to differentiate into Th1 and Th17 lymphocytes. These activated T-cells secrete cytokines that promote keratinocyte proliferation and enhance angiogenesis in the affected skin lesions (50). Building on these findings, Su *et al*. observed comparable transcriptional characteristics between psoriasis and atherosclerosis. They hypothesized that these shared mechanisms could provide valuable insights into the overlapping pathogenesis of both conditions. To explore this further, they applied sophisticated bioinformatics and enrichment analysis on two gene expression datasets (GSE30999 and GSE28829) to identify overlapping differentially expressed genes (DEGs) and their relevance in psoriasis and atherosclerosis. Their analysis identified 16 common hub genes—LYN, CSF2RB, IL1RN, RAC2, CCL5, IRF8, C1QB, MMP9, PLEK, PTPRC, FYB, BCL2A1, LCP2, CD53, NCF2, and TLR2—that were significantly overexpressed in both psoriatic skin lesions and atherosclerotic plaques. These genes were found to be primarily involved in the lipopolysaccharide-mediated signaling pathway, the cytokine-mediated signaling pathway, and the cellular response to interferon-gamma. This highlights the central role of lipopolysaccharides and cytokines in the pathogenesis of psoriasis and atherosclerosis  (51).

**Figure 3:**
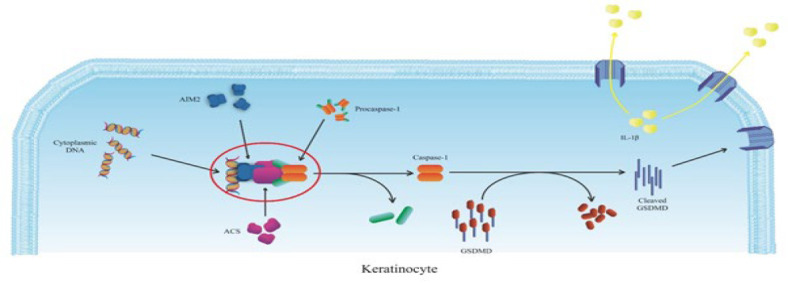
Formation of the AIM2 inflammasome complex (the red circle), resulting in the release of IL-1β from psoriatic keratinocytes

Further research has shown that inflammasomes also play a significant role in developing psoriasis (52). One such inflammasome, AIM2 (Absent in Melanoma 2), is a cytoplasmic double-stranded DNA sensor. AIM2 forms a complex with various proteins upon activation, creating the AIM2 inflammasome, which is closely associated with psoriasis (53). AIM2 expression is notably elevated in psoriatic skin, particularly in keratinocytes (54). As a member of the PYHIN family, AIM2 contains one or two HIN (hematopoietic, interferon-inducible, and nuclear) domains at its C-terminus, which are responsible for recognizing and binding cytoplasmic DNA. Additionally, its N-terminal Pyrin domain (PYD) interacts with an adapter protein called apoptosis-associated speck-like protein containing a CARD (ASC). This interaction facilitates the binding of ASC to procaspase-1, triggering its activation and cleavage into caspase-1. The active caspase-1 cleaves gasdermin-D (GSDMD), which then inserts its N-terminal domain into the cell membrane, forming pores that release cytokines. This pore formation, caused by GSDMD insertion, induces pyroptosis—a specialized form of programmed cell death—exacerbating inflammation in the affected tissues (55-58). Upon activation, procaspase-1 is cleaved into caspase-1, which cleaves gasdermin-D (GSDMD). GSDMD’s N-terminal inserts into the cell membrane, creating a pore through which cytokines can be released. This pore formation, attributed to GSDMD insertion, can induce pyroptosis, a specialized form of cell death, exacerbating inflammation (59). As shown in Figure 3, in keratinocytes affected by psoriasis, the build-up of cytoplasmic DNA activates the AIM2 inflammasome, resulting in the secretion of IL-1β and fostering T-cell-mediated skin inflammation (42, 60).

In psoriasis, the transcription factors Nuclear Factor Kappa B (NF-κB) and Aryl Hydrocarbon Receptor (AhR) play key roles in regulating inflammatory pathways (61, 62). A similar inflammatory involvement is observed in Alzheimer’s disease (AD), where several critical transcription factors, including Repressor Element 1-Silencing Transcription/Neuron-Restrictive Silencer Factor (REST/NRSF), Signal Transducer and Activator of Transcription 3 (STAT3), and Nuclear Factor Erythroid 2-Related Factor 2 (NRF2), are central to the disease’s pathophysiology. These factors influence gene regulatory networks associated with apoptosis, autophagy, and inflammatory responses, contributing to the neurodegenerative processes that characterize AD (63, 64). Notably, targeting these transcription factors has shown therapeutic promise in both psoriasis and AD, suggesting that they may play analogous roles in the pathophysiology of both conditions (65, 66). An *in silico* analysis aimed to explore the transcription factors and their upstream regulators involved in both psoriasis and AD. This study utilized gene expression datasets that included lesional and non-lesional skin samples from psoriasis patients, hippocampal tissue from AD patients, and age-matched healthy controls. Among the common differentially expressed genes (DEGs) identified in both diseases, five transcription factors—PPARG, ZFPM2, ZNF415, HLX, and ANHX—were highlighted. PPARG, ZFPM2, ZNF415, and HLX were down-regulated in psoriasis and AD, while ANHX was up-regulated. Further analysis suggested that these transcription factors interact and are involved in inflammatory processes and abnormal metabolism. Additionally, ZNF384 was identified as the key transcription factor that modulates the expression of PPARG, ZNF415, HLX, and ANHX, pointing to its potential as a therapeutic target for psoriasis and AD (67).

7SL RNA, a long non-coding RNA, is a critical component of the signal recognition particle (SRP) complex, which is involved in ribonucleoprotein-mediated processes such as intracellular protein synthesis and localization. Reduced expression of 7SL RNA has been linked to impaired antiviral responses, underscoring its importance in the host’s defense mechanisms against viral infections (68). Interestingly, 7SL RNA is significantly up-regulated in various tumors, promoting tumor cell proliferation by inhibiting the translation of p53 (69). A study examining 7SL RNA expression in lesional and perilesional healthy skin of psoriasis patients revealed a significant down-regulation of 7SL RNA in psoriatic lesions compared to adjacent healthy skin. This reduction suggests that diminished 7SL RNA expression may impair antiviral responses, potentially disrupting the skin microbiota and influencing psoriasis pathogenesis (70).

Dupilumab, a fully human monoclonal IgG4 antibody, selectively inhibits the alpha subunit of the interleukin-4 (IL-4) receptor and the interleukin-13 (IL-13) receptor (71). It has shown significant efficacy in improving outcomes for patients with moderate-to-severe atopic dermatitis who have not responded adequately to standard therapies. However, cases of psoriasis lesions have been reported in patients receiving dupilumab treatment, a phenomenon referred to as dupilumab-induced psoriatic eruption (DI-Pso) (72-74). A study comparing the skin transcriptomic profiles of patients with DI-Pso, atopic dermatitis, and plaque psoriasis found that DI-Pso lesions, compared to healthy skin, plaque psoriasis, and atopic dermatitis, exhibited activation of the T helper 17/IL-23 axis, with elevated IL-36 expression levels. In contrast, T-helper 2-related genes were down-regulated in DI-Pso compared to other conditions. Additionally, terminal differentiation markers, such as FLG and keratin genes K1 and K10, were more significantly down-regulated in DI-Pso skin lesions than atopic dermatitis and plaque psoriasis lesions, indicating potential alterations in skin barrier function. These findings suggest that the pathogenesis of DI-Pso involves a shift in immune responses from T helper 2 dominance to a polarization toward IL-36 and T helper 17 pathways, accompanied by skin barrier dysfunction (75). 

Psoriatic arthritis (PsA) is a complex inflammatory disorder that includes skin psoriasis, joint involvement (peripheral and axial), enthesitis, and dactylitis. Although IL-6 levels are elevated in the synovium of PsA patients, they are even higher in the affected skin compared to peripheral blood. Studies involving specific IL-6 inhibitors have suggested that IL-6 may not be the primary driver of joint disease in PsA (76). Sobolev *et al*. analyzed IL-6 gene expression patterns in peripheral blood mononuclear cells (PBMCs) from PsA and psoriasis patients to identify potential biomarkers for differential diagnosis. Their gene expression analysis revealed that IL-6 expression was significantly elevated in PsA (192-fold) and psoriasis (147-fold) patients compared to healthy controls. Interestingly, when subdividing psoriasis patients based on disease severity, significant increases in IL-6 gene expression were observed in severe psoriasis and PsA subgroups, suggesting that elevated IL-6 expression in PBMCs may serve as a biomarker for the progression of psoriasis to arthropathic manifestations (37).

A recent study examined the relationship between growth differentiation factor-15 (GDF-15), a transforming growth factor-β (TGF-β) superfamily member with recognized immunomodulatory roles and the severity of psoriasis. The research investigated how serum levels of GDF-15 correlated with its gene expression in patients with psoriasis. The findings revealed that serum levels of GDF-15 were higher in patients with more severe forms of psoriasis than those with milder cases. Likewise, the gene expression of GDF-15 was more significant in individuals with more severe psoriasis. These results suggest that GDF-15 could potentially serve as a valuable biomarker for assessing the severity and progression of psoriasis (77).

Another study examined the association between psoriasis and serum levels of omentin, an adipokine with anti-inflammatory properties. Omentin concentrations were significantly lower in psoriasis patients compared to healthy controls. Omentin exerts its anti-inflammatory effects by modulating the inflammatory cytokine network, inhibiting endothelial adhesion molecules and cyclooxygenase-2 (COX-2) expression, which are typically up-regulated in response to TNF-α (78). The reduced levels of omentin in psoriasis patients may suggest heightened TNF-α-related inflammation, contributing to the inflammatory processes involved in psoriasis pathogenesis. 

*Susceptibility loci and their associated polymorphisms*

Advancements in genomic research have revealed the critical role of specific genetic loci and their polymorphisms in shaping an individual’s susceptibility to psoriasis. These loci not only influence the onset of the disease but also modulate immune responses, contributing to its chronic inflammatory nature. Among the key areas of investigation are the genetic variations in cytokine families that regulate inflammation and immune function. One such family, the interferon lambda (IFN-λ) cytokine group, has emerged as a significant player in the pathogenesis of psoriasis. Research demonstrates that IFN-λ1 (IL-29) serum concentrations are significantly increased in individuals with psoriasis vulgaris compared to non-psoriatic controls. Additionally, IL-29 has been identified as a potent up-regulator of mRNA expression for key pro-inflammatory cytokines, including IL-6, IL-17, and TNF-α, in peripheral blood mononuclear cells (PBMCs) derived from psoriasis vulgaris patients (79). The association between psoriasis and specific polymorphisms in the genetic loci of IL-29 and IL-28B, both members of the IFN-λ cytokine family, has been investigated. This study assessed two single-nucleotide polymorphisms (SNPs) of IL-28B: rs12979860 (IL-28 C/T) and rs8099917 (IL-28 T/G), along with a polymorphism of IL-29, rs30461 (IL-29 T/C). The genotype and allele frequency distributions for rs12979860 (IL-28 C/T) and rs30461 (IL-29 T/C) were comparable between psoriasis patients and control subjects, with the observed differences failing to achieve statistical significance. A statistically significant difference has been identified in the TG genotype of rs8099917 when comparing the two groups, with the TG genotype correlating with a 1.9-fold elevated risk of developing psoriasis. This finding suggests a potential association of the G allele in the IL-28 rs8099917 polymorphism with the pathogenesis of psoriasis (80).

The programmed cell death protein 1 (PD-1), encoded by the PDCD1 gene, is an immune checkpoint receptor that contains an immunoreceptor tyrosine-based inhibitory motif (ITIM). This receptor is crucial in down-regulating autoreactive T-cell activity, contributing to immune tolerance, and preventing autoimmunity (81). Hua *et al*. expanded on earlier findings that suggested PD-1’s involvement in the inflammatory mechanisms of psoriasis by conducting a study focused on six functional SNPs in the PDCD1 gene. These SNPs have been previously linked to various autoimmune conditions, including systemic lupus erythematosus, rheumatoid arthritis, and multiple sclerosis. Their research aimed to determine the association between these genetic polymorphisms in PDCD1 and the pathophysiology of psoriasis. Among the six SNPs evaluated, only PD1.6 located in the 3′-untranslated region (rs10204525, G>A) demonstrated a statistically significant association with psoriasis in both genotype and allele frequency analyses. Specifically, the heterozygous AG genotype was linked to an increased risk of developing psoriasis compared to the homozygous AA genotype. Furthermore, the combined AG and GG genotypes also showed a notable association with heightened psoriasis risk. Regarding allele analysis, individuals carrying the G allele exhibited an odds ratio of 1.65 relative to those with the A allele, indicating that the presence of the G allele in the PD1.6 polymorphism is associated with a greater susceptibility to psoriasis. It is important to note that this polymorphism does not correlate with the severity of the disease (82).

The PTPN2 gene encodes a T-cell-specific protein that functions as a negative regulator of signaling pathways, contributing to the modulation of immune responses (83). This molecule regulates multiple signaling cascades and mechanisms, notably the JAK/STAT pathway, which plays a crucial role in T cell-mediated immunological responses (84). Mei *et al*. conducted a study to assess the relationship between polymorphisms in the PTPN2 gene and the susceptibility to psoriasis within the Northeastern Chinese population. This investigation was grounded in the gene’s established role in predispositions to autoimmune conditions, particularly type 1 diabetes and Crohn’s disease. The researchers genotyped 13 single nucleotide polymorphisms (SNPs) in PTPN2. Their results revealed a significant prevalence of the rs2847297-G allele, rs67555-C allele, and rs482160-A allele among psoriasis cases compared to controls. These findings suggest a potential link between these specific alleles and an increased risk of developing psoriasis (85).

Signaling transducers and activators of transcription (STATs) are critical transcription factors that modulate gene expression by binding to specific DNA consensus sequences. These proteins are pivotal in intracellular signaling pathways, translating extracellular signals into transcriptional responses that govern various physiological processes (86). Among them, STAT3 has been implicated in the downstream signaling of cytokines such as IL-6 and IL-10, contributing to innate immune responses in psoriatic epidermis (87). Activation of STAT4 can lead to specific Th1 differentiation, potentially intensifying responses to interferon (IFN)-γ and triggering psoriasis (88). Zhou *et al*. investigated the relationship between STAT gene polymorphisms and psoriasis, focusing on the northeastern Chinese population, given the established roles of STAT3 and STAT4 in autoimmune pathogenesis. The study involved genotyping multiple single nucleotide polymorphisms (SNPs) within the STAT3 locus, specifically rs2293152, rs3816769, rs4796793, and rs744166, as well as SNPs rs7574865 and rs3024866 located in the STAT4 gene. The prevalence of the rs744166 AA genotype of the STAT3 gene was significantly reduced in the psoriasis cohort compared to the control group. In contrast, the frequencies of the GG genotype and G allele were considerably elevated in the psoriasis cases, indicating a potential association of the minor allele G with heightened susceptibility to psoriasis. Regarding the STAT4 gene, the rs7574865 GG genotype was less frequently observed in the psoriasis cases, suggesting it may act as a protective factor against the disease. Conversely, the GT and TT genotypes, along with the T allele, were significantly more prevalent in the psoriasis patients than in healthy controls, implying that these genotypes confer an increased risk for psoriasis development (89).

Msafiri Makene *et al*. (90) explored the relationship between polymorphisms of the CARD14 gene (caspase recruitment domain family member 14) and psoriasis vulgaris in the Southern Chinese Han ethnic group. CARD14 was chosen based on insights derived from genome-wide association studies (GWAS) and focused candidate gene analyses, which have identified several single nucleotide polymorphisms (SNPs) associated with increased susceptibility to psoriasis. Notably, a significant proportion of genes related to innate immunity, including CARD14, are part of the NF-κB pathway, crucial in sustaining inflammation in chronic psoriasis. A total of 32 polymorphisms were identified, with 3 SNPs demonstrating significant associations. Specifically, one of these SNPs is exonic (rs144475004), while the other two are intronic (rs4889836 and rs4889989).

Bioinformatics analysis indicated that the variant rs144475004 within the exon led to an amino acid substitution from aspartate to histidine. Furthermore, when evaluated under co-dominant and recessive genetic models, the rs4889836 T/C polymorphism demonstrated a significant association with psoriasis. In contrast, the variant rs4889989 exhibited a significant relationship with psoriasis solely under the dominant genetic model. Additionally, the study identified two protective haplotypes, termed CARD14-1 and CARD14-2, associated with a reduced risk of developing the disease. Their frequencies were 1.49% and 6.96% in cases and healthy controls, respectively. Table 1 outlines the characteristics of these haplotypes.

The results highlighted a significant correlation between the CARD14 gene and the predisposition to psoriasis vulgaris in the Han population.

Oka *et al*. conducted a study to replicate findings from psoriasis-related GWAS, focusing on polymorphisms within the IL12B and IL23R genes. The IL12B gene encodes the shared IL-12p40 subunit found in the cytokines IL-12 and IL-23, while IL23R encodes a component of the IL-23 receptor. Prior research has established these genes as genetic susceptibility factors for psoriasis based on candidate gene studies and genome-wide association studies in Chinese and European populations. In their study, the researchers genotyped two single nucleotide polymorphisms (SNPs) in the IL12B gene and one SNP (rs11209026) in the IL23R gene. Their analysis revealed significant associations between psoriasis and the two IL12B SNPs, specifically rs3212227 and rs6887695. Conversely, no notable association was identified between psoriasis and the IL23R SNP, rs11209026. Furthermore, the study uncovered a significant association with a protective haplotype characterized by the C-C genotype in the IL12B gene within the Japanese population (91).

The association between *IL12B* gene polymorphisms and psoriasis susceptibility has also been reported in an Egyptian cohort study, in which the rs610604 in the *IL12B* gene exhibited a highly significant association with psoriasis in patients with early onset of the disease (less than 30 years) (92). This finding suggests that the presence of associations between IL12B polymorphisms and psoriasis across subjects from diverse ethnic backgrounds underscores the significance of this gene in the pathogenesis of the disease.

A comprehensive meta-analysis investigated three specific polymorphisms within the ERAP1 gene locus, specifically rs26653, rs30187, and rs27524 (93). *ERAP1* plays a pivotal role in processing antigenic peptides before loading them onto HLA-I molecules for antigen presentation (94), and previous studies have reported this gene and its polymorphisms to be implicated in psoriasis susceptibility (95, 96). In alignment with prior research, the meta-analysis corroborated that the T allele of the rs30187 T/C polymorphism, along with the A allele of the rs27524 A/G polymorphism, exhibited a significant association with the susceptibility to psoriasis.

A study on the Turkish population explored the potential correlation between the rs2236242 polymorphism in the vaspin gene, a serine protease inhibitor derived from visceral adipose tissue, and the risk of psoriasis. The results demonstrated a significantly reduced prevalence of the rs2236242 TT genotype in psoriasis patients relative to control subjects. Additionally, individuals with the TA genotype exhibited a 2.38-fold higher risk of developing psoriasis than those with the TT genotype. In contrast, no substantial differences were identified between the AA and TT genotypes in this context (97).

In an Egyptian case-control study, researchers evaluated S100A8 serum levels and the S100A8 rs3806232 polymorphism to assess their potential association with psoriasis susceptibility and severity. The study revealed a significant elevation of serum S100A8 levels in patients with psoriasis compared to control subjects, alongside a positive correlation with disease severity. Notably, the rs3806232 AA genotype and A allele were markedly more frequent among individuals with psoriasis, indicating an increased susceptibility to the disease relative to the AG, GG, and G alleles. Additionally, the AA genotype demonstrated a significant association with the severity of psoriasis and elevated S100A8 serum concentrations (98).

A related study investigating the role of forkhead box class O3A (FOXO3a), a transcription factor crucial for intercellular regulation, oxidative stress response, DNA repair mechanisms, and apoptosis, has revealed significantly elevated serum levels of FOXO3a in patients with psoriasis when compared to healthy controls. Notably, serum FOXO3a concentrations were markedly higher in individuals with severe psoriasis than in those with mild-to-moderate forms of the disease. Furthermore, genotypic analysis of the FOXO3a rs13217795 polymorphism showed a significantly increased prevalence of the homozygous TT genotype and the T allele within the psoriasis cohort relative to the control group (99). 

Overall, susceptibility alleles in genetic loci that exhibit association with a specific condition have the potential to serve as predictive and/or prognostic markers. This feature could be of considerable importance, especially in multifactorial disorders in which a combination of genetic and environmental phenomena additively predisposes an individual to the disease. This importance is reflected by the fact that for individuals carrying highly associated alleles in previously established susceptibility loci, limiting the influence of environmental factors, such as adjusting lifestyle, might significantly affect the prevention of the disease in question. The same scenario is applied to psoriasis, given that it is also a multifactorial condition. Concerning the genotype-phenotype correlation associated with specific alleles linked to a particular condition, the causality may be directly attributable to the polymorphism itself. This is evident when a single nucleotide polymorphism (SNP) within the reading frame alters the amino acid sequence of the respective protein. Additionally, variations located in the promoter or other regulatory regions can influence the expression levels of the gene product. Alternatively, an apparent association with a polymorphism may simply occur due to its proximity to another locus responsible for a portion of the genetic contribution to the disease. Either way, uncovering the genetic loci associated with a disorder might significantly impact its management and shed light on the mechanisms underlying its development.

## Conclusion

Psoriasis is a chronic inflammatory dermatosis characterized by dysregulated immune responses and hyperproliferation of keratinocytes. Both heritable genetic predispositions and various environmental factors contribute to its pathogenesis, highlighting the complexity of this condition as a public health concern. The prevalence of psoriasis significantly impacts a considerable segment of the global population, with substantial ramifications for patients’ physical health, social interactions, and psychological well-being. This review highlights the multifactorial nature of psoriasis, detailing the contribution of numerous genetic loci to its progression and severity. Identifying these genetic factors can aid in developing personalized treatment plans that target the underlying cause of the disease, improving patients’ outcomes and enhancing their quality of life. 

**Table 1:** Characteristics of the protective haplotypes against psoriasis in the CARD14 gene (90)

	SNPs	Haplotype	Patients	Healthy individuals	*P*-value
CARD14-1	rs4889989, rs2289539, rs144475004	GTC	3	11	0.01
CARD14-2	rs144475004, rs4889990, rs3829612, rs532775553, rs3813064	CGCGC	3	11	0.01

## Declaration

We acknowledge the use of ChatGPT to correct grammatical errors.
